# Protein-ligand binding region prediction (PLB-SAVE) based on geometric features and CUDA acceleration

**DOI:** 10.1186/1471-2105-14-S4-S4

**Published:** 2013-03-08

**Authors:** Ying-Tsang Lo, Hsin-Wei Wang, Tun-Wen Pai, Wen-Shoung Tzou, Hui-Huang Hsu, Hao-Teng Chang

**Affiliations:** 1 Department of Computer Science and Engineering, National Taiwan Ocean University, Keelung, Taiwan, R.O.C; 2 Department of Life Sciences, National Taiwan Ocean University, Keelung, Taiwan, R.O.C; 3 Center of Excellence for Marine Bioenvironment and Biotechnology, National Taiwan Ocean University, Keelung, Taiwan, R.O.C; 4 Department of Computer Science and Information Engineering, Tamkang University, New Taipei City, Taiwan, R.O.C; 5 Graduate Institute of Molecular Systems Biomedicine, China Medical University, Taichung, Taiwan, R.O.C; 6 China Medical University Hospital, Taichung, Taiwan, R.O.C

## Abstract

**Background:**

Protein-ligand interactions are key processes in triggering and controlling biological functions within cells. Prediction of protein binding regions on the protein surface assists in understanding the mechanisms and principles of molecular recognition. *In silico *geometrical shape analysis plays a primary step in analyzing the spatial characteristics of protein binding regions and facilitates applications of bioinformatics in drug discovery and design. Here, we describe the novel software, PLB-SAVE, which uses parallel processing technology and is ideally suited to extract the geometrical construct of solid angles from surface atoms. Representative clusters and corresponding anchors were identified from all surface elements and were assigned according to the ranking of their solid angles. In addition, cavity depth indicators were obtained by proportional transformation of solid angles and cavity volumes were calculated by scanning multiple directional vectors within each selected cavity. Both depth and volume characteristics were combined with various weighting coefficients to rank predicted potential binding regions.

**Results:**

Two test datasets from LigASite, each containing 388 bound and unbound structures, were used to predict binding regions using PLB-SAVE and two well-known prediction systems, SiteHound and MetaPocket2.0 (MPK2). PLB-SAVE outperformed the other programs with accuracy rates of 94.3% for unbound proteins and 95.5% for bound proteins via a tenfold cross-validation process. Additionally, because the parallel processing architecture was designed to enhance the computational efficiency, we obtained an average of 160-fold increase in computational time.

**Conclusions:**

*In silico *binding region prediction is considered the initial stage in structure-based drug design. To improve the efficacy of biological experiments for drug development, we developed PLB-SAVE, which uses only geometrical features of proteins and achieves a good overall performance for protein-ligand binding region prediction. Based on the same approach and rationale, this method can also be applied to predict carbohydrate-antibody interactions for further design and development of carbohydrate-based vaccines. PLB-SAVE is available at http://save.cs.ntou.edu.tw.

## Background

The study of protein binding site prediction assists in understanding the mechanisms and principles of molecular recognition, provides information for drug design and vaccine development, and enables more detailed annotation of function in protein databases and in the construction of visual displays of protein-protein interaction networks [1,2]. In recent years, various *in silico *methods for prediction of protein-protein and protein-ligand binding sites have been developed [3], but as the number of known protein structures and protein-complex structures has grown exponentially in the last decade, a fast and effective algorithm to identify binding regions of a protein is still urgently needed. An especially important application is carbohydrate vaccine development. This has gained much attention in recent years as a new strategy against pathogen infection and cancers, and the prediction of binding pockets between a glycan and antibody could be very valuable in the development of carbohydrate-based therapeutics [1]. The binding affinity of a carbohydrate-based antibody is normally weaker than that of a protein-based antibody. A tool for predicting properties of carbohydrate binding sites could therefore provide sufficient information for the development of carbohydrate-based vaccines. Historically, several different approaches based on geometric characteristics, physicochemical properties, or combinations of these have been used to predict regions of protein interaction. For example, an algorithm using surface complementarity, calculated from the Connolly surfaces and geometric characteristics of proteins, has been used to model protein-protein interactions [4], and physical shape characteristics are frequently used to analyze and identify surface interfaces such as accessible surface areas [5,6], sequence conservation [7,8], and amino acid composition [9]. In addition, a number of different approaches have used Fourier-based concepts, transforming a three-dimensional grid onto a set of orthogonal basis functions, and calculating overlapping areas using Fast Fourier Transform techniques [10-12]. Another approach is to consider the physicochemical properties of interface residues using statistical methods to predict binding sites. For example, aliphatic and aromatic residues are found at interface regions at a higher frequency compared with charged residues, and several methods have exploited this observation by examining the specific composition of amino acids in surface regions to predict binding sites [13-15]. Although most previous methods for predicting protein binding regions have adopted similar approaches for analyzing protein-protein interfaces and protein-ligand binding regions, these two major types of binding exhibit different characteristics such as binding architecture and binding region size [16]. Here, we designed an improved prediction system for protein-ligand binding, in which the query proteins are assumed to be rigid and their geometric characteristics such as solid angle, cavity depth, and volume are considered. In keeping with most existing algorithms, we also used shape complementary as the primary filter to rank all potential binding regions. In addition, we considered a grid-based construction of structure for surface residue identification and used parallel processing mechanisms for more efficient computation on geometric features. Thus, irregularly shaped cavities and pockets on the protein surface can be efficiently identified and placed in a rank order of potential protein-ligand binding regions.

In our approach, we used the concept of the solid angle and its associated features as the main geometric attributes for analysis of protein-ligand binding potential. Connolly proposed the solid angle approach to examine protein surface binding characteristics such that if two three-dimensional shapes fit together, then the sum of their two solid angles equals 4π in three-dimensional space [17]. There are two main methods for computing solid angles: the first approach uses the Gauss-Bonnet theorem to find solid angles subtended by surface regions; the second approach calculates the steradian formed by a virtual sphere on the protein surface, and then divides this by the square of the radius of the virtual sphere. Both methods calculate the solid angle of a specified surface region. Several researchers adopted the solid angle approach, and valuable results have been published in the fields of protein docking [18,19] and structure alignment [20]. Due to the huge number of atoms on a protein surface and the resulting demand on computational power and time for solid angle calculations, we used Compute Unified Device Architecture (CUDA) technology (NVIDIA Corporation, Santa Clara, CA) to enhance execution speed of the proposed algorithms. CUDA is a parallel computing architecture that utilizes graphics processing units (GPUs) for general-purpose computing. GPUs were originally employed to speed up graphics display and could quickly and easily generate multiple threads. In addition, floating point operations and memory bandwidth performance are much faster with GPUs than with central processing units (CPUs), as the multi-core architecture allows each thread to perform an identical computing task simultaneously [21]. Since the introduction of CUDA in 2007, harnessing the power of the GPU has become easier, and recently, numerous GPU-based algorithms have been proposed in bioinformatics for sequence alignment [21-24], protein docking [25], surface area calculations [26,27], molecular dynamic simulations [28], and in systems biology [29]. Here, we use CUDA architecture to reduce computational time and develop an effective prediction system to identify binding regions by evaluating the geometric features of solid angle, depth, and volume of a cavity on a protein surface. Based on performance comparisons with other methods and validation of the predictions via experimental data, our algorithm, PLB-SAVE, is effective for detecting protein-ligand binding regions, and we believe it has considerable potential in drug and vaccine development.

## Methods

**Figure 1:**
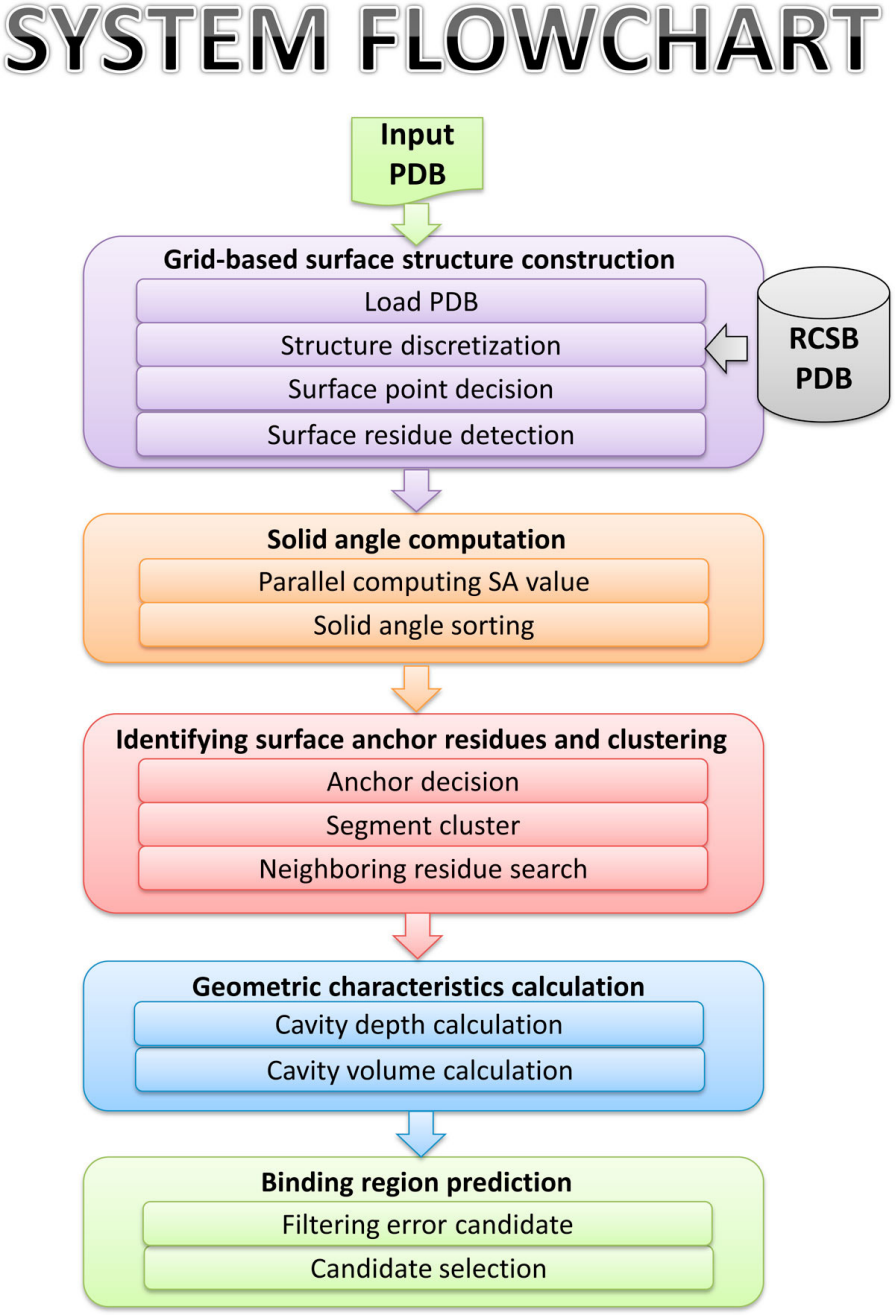
**Schematic representation of the various components of PLB-SAVE**.

The PLB-SAVE algorithm involves five main steps (Figure 1), starting with importing a Protein Data Bank (PDB, http://www.rcsb.org/pdb/home/home.do) file for analysis. Multiple chains or single chain of a protein structure can be evaluated according to user's requirements. The CUDA architecture, developed for parallel computing for graphics processing, can handle the spatial features of hundreds of thousands of atoms in the protein surface of the query protein. Each step in the algorithm is briefly described below.

### Grid-based surface structure construction

The imported protein structure file, in PDB format [30], contains complete spatial coordinate information obtained by X-ray crystallography, NMR spectroscopy, cryo-electron microscopy, or *in silico *prediction methods. In this step, the coordinates of atoms and their corresponding van der Waals radii are transformed into corresponding volumetric pixels (voxels) within a grid structure. This facilitates rapid identification of protein surfaces and allows efficient calculation of solid angles for each atom. After discretization processes, the query protein is represented as a set of discrete voxels that are categorized as inside (buried) or outside (surface) portions of the query protein.

### Solid angle computation

**Figure 2:**
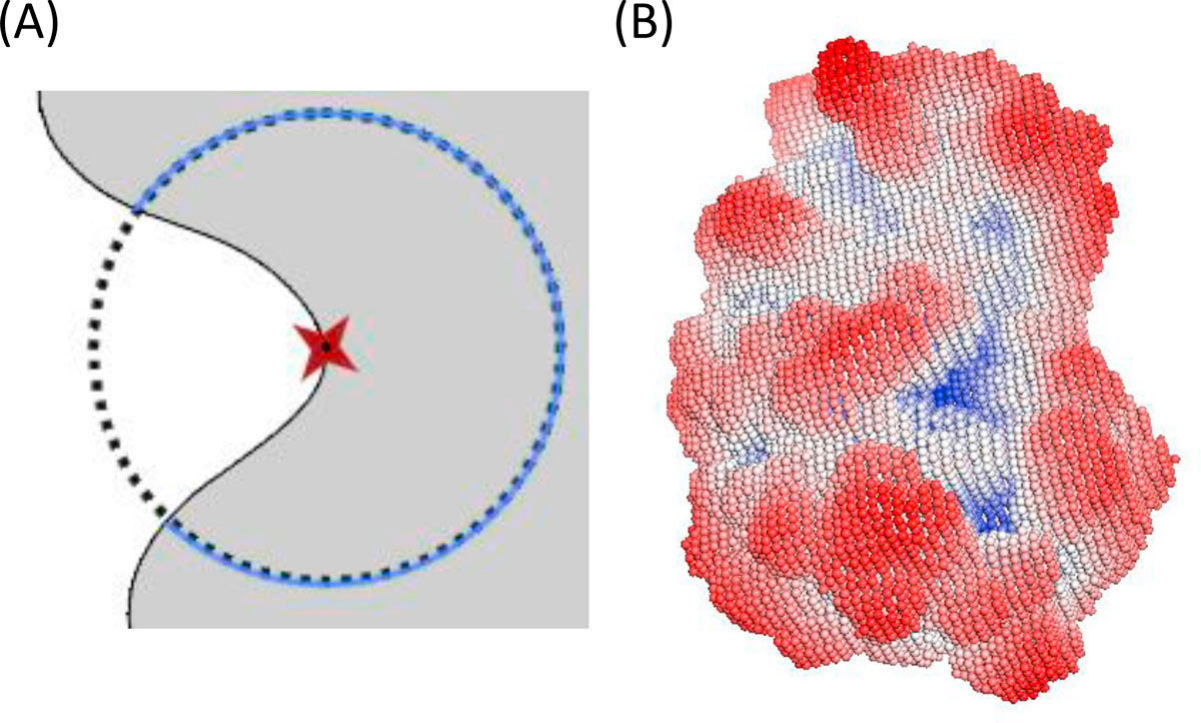
**Illustration of solid angle calculation**. (A) A 2D representation of solid angle calculation, where *V_in _*is the volume of the virtual sphere located within the interior regions of the query protein (blue circles), and *V_sphere _*represents the volume of the total sphere (black circles). (B) Calculated solid angles on the surface area of the query protein (PDB ID: 1TPA). Red spheres are recognized as protruding regions, white or lighter-shaded spheres represent flat regions, and blue spheres represent concave regions on the protein surface.

For each surface voxel within a protein, the PLB-SAVE algorithm computes its corresponding solid angle as shown in Equation 1:

(1) $$SA\left(V_{i}\right)=\left(V_{in}/V_{sphere}\right)*4\pi$$

where *SA*(*v_i_*) is the solid angle of the surface voxel *v_i _*, *V_in_* denotes the number of overlapped voxels between the previously defined virtual sphere centered at *v_i _*and the query protein, and *V_sphere_* denotes the total number of voxels located within the identical virtual sphere. In this step, the recommended radius of the virtual sphere is 6 Å as Connolly's suggestion for all surface voxels [17]. PLB-SAVE uses CUDA coding modules to compute solid angles on all surface voxels in parallel to enhance the computational performance. Figure 2(A) illustrates how to efficiently approach a solid angle from Equation 1, and an example of solid angle distributions for all surface voxels of the query protein is shown in Figure 2(B). The red dots represent surface voxels with small values of solid angle, and these surface voxels are generally expressed as voxels located on convex regions; in contrast, the blue dots represent surface voxels with relatively large values of solid angle on the protein surface, and these surface voxels occurred in concave areas. Relatively flat regions (i.e., neither concave nor convex) are represented by white or light grey dots when the value of solid angles is near 2π.

### Identifying surface anchor residues and clustering

Because we are trying to identify binding cavities in the query protein, only those surface voxels possessing solid angles in the highest 20% were clustered into representative groups in order. Two surface voxels would be clustered into the same group if they are neighboring voxels located within a threshold distance of 20 Å and both voxels have high solid angles at a similar level. The surface voxel with the largest solid angle within the selected cluster is deemed the representative anchor for the group.

**Figure 3:**
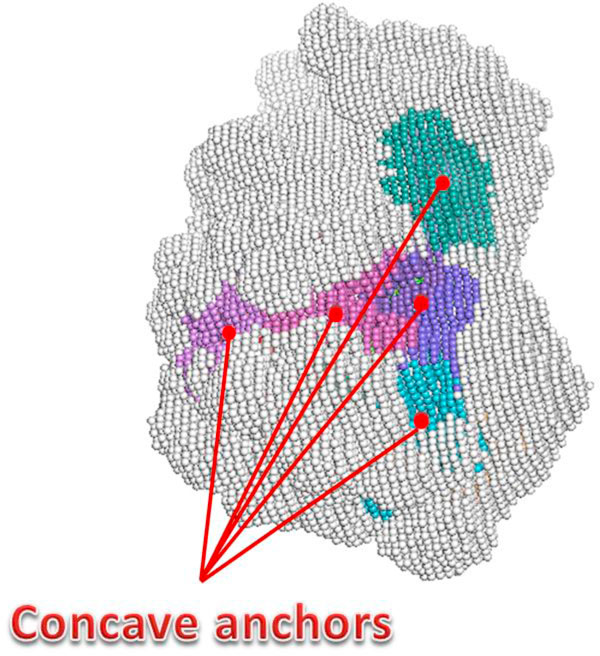
**An example of clustered pocket regions**. The clustered surface residue groups from the Anhydrotrypsin protein (PDB ID: 1TPA) according to the ranking of solid angles. Three indicated red dots denote the representative anchors of the clustered concave pockets. Dark grey clusters represent the clustered convex regions.

Figure 3 shows an example of the surface voxels after clustering processes. The different colors represent clustered groups, and the three indicated red dots denote the anchors for these groups. These identified groups generally possess greater average solid angles (concave regions), and they are stored separately to facilitate future applications on identification of binding regions.

### Geometric characteristics calculation

After the assignment of clustered groups and representative anchors, the algorithm calculates additional geometric characteristics for each group, including cavity depth and volume of the identified anchor regions. These selected characteristics are required to be rotation- and translation-invariant, and most importantly, must be feasible and efficient for protein-ligand binding analysis. The efficacious geometric characteristics are described below.

#### Cavity depth calculation

**Figure 4:**
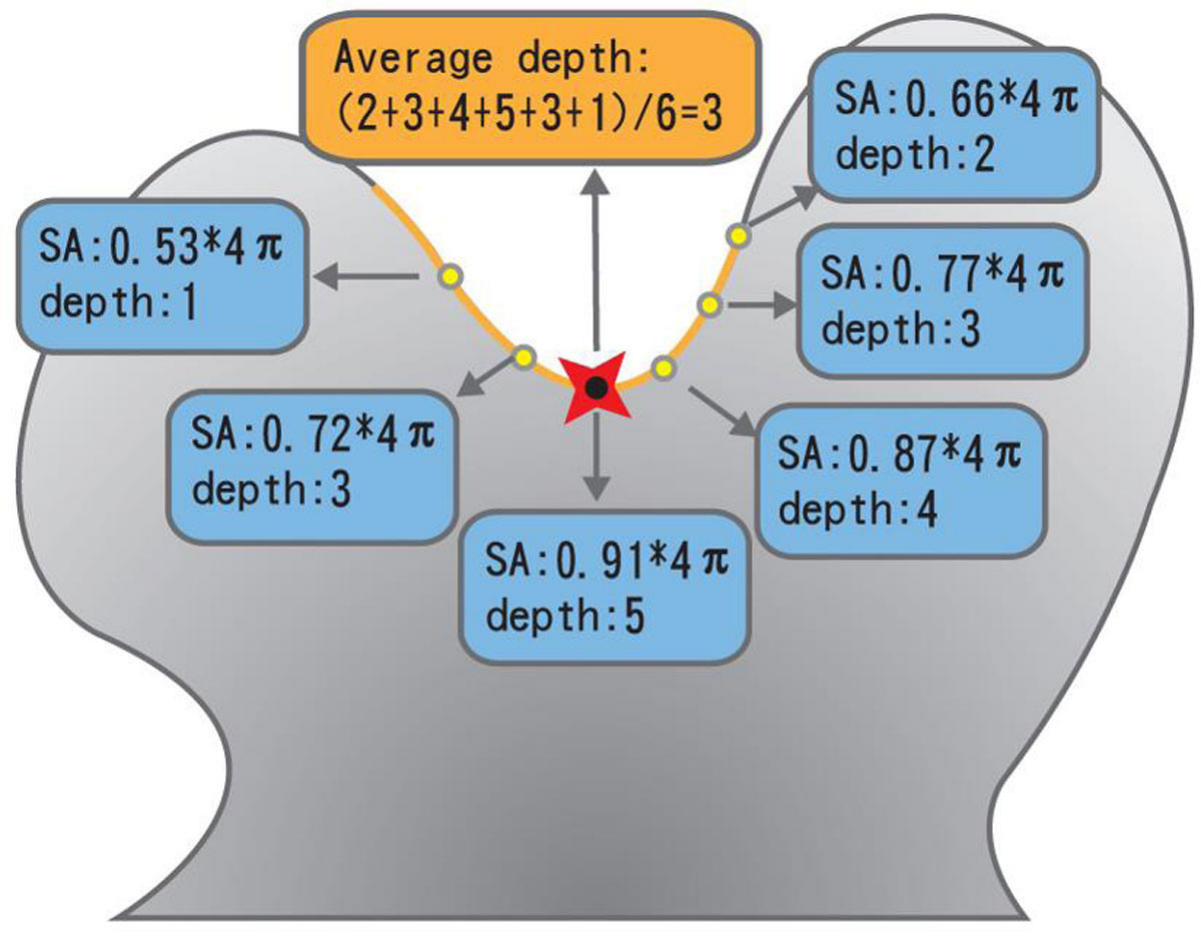
**An example of an average depth indicator for an identified cavity with six neighboring surface residues**.

Although a defined surface anchor may have a large solid angle, it is not a necessary condition for all of its neighboring surface elements, and we found that a cluster of surface elements containing different levels of solid angles sometimes caused incorrect binding region prediction. To avoid such large variations of neighboring surface elements within a group, an enhanced feature of average depth of a potential cavity was calculated and verified. The proposed average depth was heuristically defined and evaluated according to Equation 2,

(2) $$Depth\left(v_{i}\right)=\left[\begin{array}{ccc} 5 & \text{if} & \text{SA}\left(v_{i}\right)\;>\;0.9{\;}^{*}\;4\pi \\ 4 & \text{if} & 0.8{\;}^{*}\;4\pi \;<\;\text{SA}\left(v_{i}\right)\;\leq 0.9{\;}^{*}\;4\pi \\ 3 & \text{if} & 0.7{\;}^{*}\;4\pi \;<\;\text{SA}\left(v_{i}\right)\;\leq 0.8{\;}^{*}\;4\pi \\ 2 & \text{if} & 0.6{\;}^{*}\;4\pi \;<\;\text{SA}\left(v_{i}\right)\;\leq 0.7{\;}^{*}\;4\pi \\ 1 & \text{if} & 0.5{\;}^{*}\;4\pi \;<\;\text{SA}\left(v_{i}\right)\;\leq 0.6{\;}^{*}\;4\pi \\ -1 & \text{else} & \end{array}\right)$$

where *Depth*(*v_i_*) denotes the transformed depth of voxel *v_i _*in the clustered group, and *SA*(*v_i_*) refers to the solid angle of *v_i_*. The simple proportional transformation from solid angles to depth indicators is designed mainly due to the observations that a surface voxel locates at the deeper position of a cavity often possessing a higher solid angle. An example with six surface voxels within a cluster is illustrated in Figure 4, in which the corresponding depth indicator of a clustered group was obtained by averaging the transformed values between solid angles and mapped depth values.

#### Cavity volume calculation

**Figure 5:**
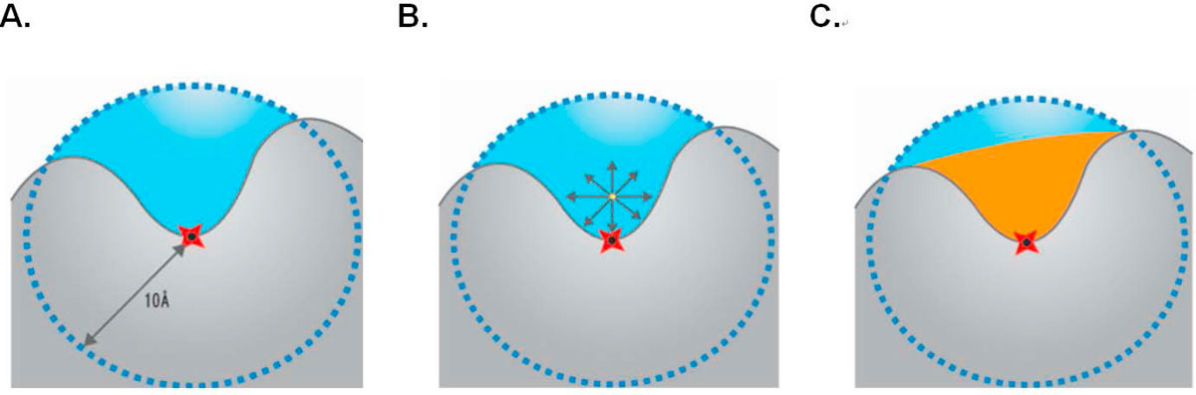
**An example of a volume indicator**. (A) A virtual sphere of 10 Å located at the center of anchor residue was constructed to evaluate the total number of potential volume voxels. (B) Seven extended directional vectors of the candidate volume voxel. (C) If a voxel possesses more than or equal to four extended directional vectors intersecting with the protein, the voxel was defined as one of the volume voxels and represented in orange. Voxels within the virtual sphere but not belonging to the volume content are depicted in blue.

The volume of selected cavities provides identifiable discrimination between binding and non-binding regions. Here, the volume indicator of a cluster is obtained by taking the anchor surface voxel as the center and surrounding it with a virtual sphere of radius 10 Å. Those voxels located within the virtual sphere, but not inside the query protein, are verified individually to see whether these voxels belonging to part of the volumetric portion within the cavity. Each voxel is considered as a virtual origin of a Cartesian coordinate system, and it is evaluated by taking seven directional vectors, including three bi-direction vectors codirectional with the x, y, and z axes and four bi-direction diagonal vectors passing through the virtual origin. If by extending a directional vector in both directions and the query protein is intersected in both directions, then this directional vector is assigned as an interior directional vector. If a given voxel possesses more than or equal to four verified interior directional vectors, then that voxel is defined as part of the volume within the cavity[31]. After examining all voxels in the virtual sphere, the total number of interior voxels gives the volume value for the cluster. An example is shown in Figure 5, where each interior voxel was evaluated and verified by this method.

### Binding region prediction

A measuring score combining linear weighting coefficients was then used to rank all identified potential binding regions, according to Equation 3.

(3) $$\text{RV}\left(v_{i}\right)=\frac{\text{CD}{\left(v_{i}\right)}_{avg}}{\text{C}{\text{D}}_{\text{max}}}\times {\text{w}}_{1}+\frac{\text{CV}\left(v_{i}\right)}{\text{C}{\text{V}}_{\text{max}}}\times {\text{w}}_{2}$$

RV(*v_i _*) is the ranked value for anchor voxel *v_i_*, CD(*v_i_*)_avg _is the value of average depth for *v_i_*, CD_max _is the maximum depth of the query protein, CV(*v_i_*) is the volume of *v_i_*, CV_max _is the maximum volume of the query protein, and the sum of both weighting coefficients, ${\text{w}}_{1}$ and ${\text{w}}_{2}$, is equal to 1.

### Parallel computing architecture by CUDA

The CUDA Toolkit, version 4.0 (Nvidia Corporation) and Visual Studio 2010 (Microsoft Corporation, Redmond, WA) were used to implement PLB-SAVE on an Intel^® ^Core^™ ^i7-2600 Processor operating at 3.40 GHz, with a 16 GB DDR3 memory and a GeForce GTX 580 graphics card (Nvidia Corporation) using the Microsoft Windows 7 operating system. In order to compare performance, PLB-SAVE was implemented onto two platforms: one with CPU architecture alone, and another with CUDA-computing architecture. Two datasets contain various sizes of proteins will be evaluated through two different computing architectures individually.

## Results and discussion

### Experimental datasets and measurements

The protein structure datasets used for testing included two types of bound and unbound proteins, collected from LigASite version 9.5 (http://www.bigre.ulb.ac.be/Users/benoit/LigASite/index.php) [32]. Each dataset contained 388 representative and non-redundant protein structures, and the binding sites of each protein were also provided for method validation. Five evaluation parameters were calculated to compare the performance with other prediction systems, including sensitivity, specificity, accuracy, positive predictive value (PPV), and Matthew's correlation coefficient (MCC). These parameters were calculated using the following equations:

Sensitivity = $\frac{\text{TP}}{\text{TP}+\text{FN}}$

Specificity = $\frac{\text{TN}}{\text{TN}+\text{FP}}$

Accuracy = $\frac{\text{TP}+\text{TN}}{\text{TP}+\text{FP}+\text{TN}+\text{FN}}$

PPV = $\frac{\text{TP}}{\text{TP}+\text{FP}}$

MCC = $\frac{\text{TP}\times \text{TN}-\text{FP}\times \text{FN}}{\sqrt{\left(\text{TP}+\text{FP}\right)\left(\text{TP}+\text{FN}\right)\left(\text{TN}+\text{FP}\right)\left(\text{TN}+\text{FN}\right)}}$

where TP is the number of true binding sites correctly predicted by our system to be binding sites; FP is the number of non-binding sites incorrectly predicted to be binding sites; TN is the number of non-binding sites correctly predicted not binding sites; FN is the number of true binding sites incorrectly predicted as non-binding sites. In this study, if the top 1 to top 3 predicted binding regions are indeed located at the true binding pocket sites, the prediction is claimed as a successful trial and the numbers of predicted binding and non-binding sites will be applied to evaluate all measurements.

### Performance of PLB-SAVE

The algorithm described here, PLB-SAVE, is freely available at http://save.cs.ntou.edu.tw. Its prediction performance was evaluated under a tenfold cross-validation scheme. Both bound (HOLO) and unbound (APO) protein sets, each containing 388 representative proteins, were randomly partitioned into ten subsets. Each partitioned subset was retained as the group of validation proteins used for evaluating the prediction model, and the remaining nine subsets were then used as the training dataset for finding the best default parameters. The cross-validation process was repeated ten times, and each of the ten subsets was used exactly once as the validation subset. Final measurements were obtained by taking the average from individual ten prediction results and the final prediction results are shown in Table 1. Both prediction performances achieved stable and superior performance compared to most previously published systems, and the performance on the bound dataset was generally better than on the unbound dataset for all measurements. This is mainly because some testing proteins in bound conditions possessing cavities with preferred and suitable structural conformations than unbound conditions.

**Table 1:** Performance of PLB-SAVE evaluated under tenfold cross-validation.

PLB-SAVE Cross-validation	APO-388 Proteins	HOLO-388 Proteins
Sensitivity	**0.579**	**0.643**

Specificity	**0.972**	**0.976**

Accuracy	**0.943**	**0.955**

PPV	**0.635**	**0.652**

MCC	**0.566**	**0.613**

To demonstrate the superior performance of PLB-SAVE, we compared the prediction results with two existing methods: SiteHound [33] and MetaPocket v2.0 (MPK2) [34]. SiteHound identified ligand binding sites by computing the interactions between a chemical probe and a protein structure, and it used the profiles of the affinity map and total interaction energy to rank predicted binding sites. MPK2 integrated eight approaches including LIGSITE^CSC ^[31], PASS [35], QsiteFinder [36], SURFNET [37], Fpocket [38], GHECOM[39], ConCavity [40], and POCASA [41], and combined predicted pocket sites from eight methods through consensus pocket analysis to improve the prediction success rate.

The aforementioned bound and unbound proteins in the two testing datasets were uploaded one-by-one to these two prediction systems, and the resulting performances are shown in Table 2 and Table 3. Although PLB-SAVE successfully predicted all 388 protein structures, only partial proteins were successfully predicted by either SiteHound or MPK2 using their on-line implementation under a limited time frame (10 minutes). Thus, to compare like with like, we selected only identical structures that were able to be individually processed by these two systems. Table 2 compares the prediction measurements from the APO dataset for 373 proteins analyzed by SiteHound and 342 proteins analyzed by MPK2 with those of PLB-SAVE. Apart from the sensitivity for the 342 proteins, which was worse than for MPK2(71.9%), all other measurements were higher using PLB-SAVE than using the other two algorithms, and the overall accuracy rate of PLB-SAVE (92.9%) was higher than for MPK2(89.9%). Similarly, for bound proteins in the HOLO dataset, PLB-SAVE successfully predicted all 388 entries, but only 374 and 339 proteins were correctly predicted by SiteHound and MPK2, respectively. Table 3(a) shows that PLB-SAVE performed better than SiteHound in terms of sensitivity, specificity, accuracy, PPV, and MCC for the 374 bound proteins. Table 3(b) shows that the average prediction results of PLB-SAVE were also better than MPK2 in most aspects, except for the sensitivity measurement, which were lower for these 339 protein structures. However, the overall accuracy rate of PLB-SAVE is 94.9% which was much higher than MPK2 of 87.0%. In addition, as previously noted, the performance of all three prediction systems for bound proteins was generally better than for the unbound proteins, due to the lower flexibility in the protein surface conformation of the bound protein, and perhaps also lower static energy. Interestingly, we found that the performance of PLB-SAVE is more consistent than SiteHound and MPK2 regarding bound and unbound protein structures. For example, prediction results performed by each software package for unbound versus bound protein led to increased performance, as judged by improved sensitivity, by 11%, 42%, and 12% for PLB-SAVE, SiteHound, and MPK2, respectively. Stable performance of a prediction system is important because the practical applications for unknown protein binding site prediction would mainly be unbound structures. Thus, the performance of PLB-SAVE showed that simple and reliable geometric features could provide a stable performance for protein binding region analysis.

**Table 2:** Prediction results of PLB-SAVE, SiteHound, and MPK2 using the APO dataset (388 proteins).

APO dataset	PLB-SAVE (373 proteins)	SiteHound (373 proteins)
Sensitivity	**0.527**	0.379

Specificity	**0.968**	0.955

Accuracy	**0.934**	0.912

PPV	**0.583**	0.399

MCC	**0.509**	0.332

(a)		

**APO dataset**	**PLB-SAVE (342 proteins)**	**MPK2 (342 proteins)**

Sensitivity	0.534	**0.719**

Specificity	**0.965**	0.918

Accuracy	**0.929**	0.899

PPV	**0.585**	0.436

MCC	**0.511**	0.496
(b)		


Calculations were performed only on proteins whose binding regions had been successfully predicted by SiteHound and MPK2. Numbers in bold indicate which software gave the better performance for each parameter. (a) PLB-SAVE compared with SiteHound using 373 proteins. (b) PLB-SAVE compared with MPK2 with using 342 proteins.

**Table 3:** Prediction results of PLB-SAVE, SiteHound and MPK2 using the HOLO dataset.

HOLO Dataset	PLB-SAVE (374 proteins)	SiteHound (374 proteins)
Sensitivity	**0.623**	0.538

Specificity	**0.975**	**0.975**

Accuracy	**0.953**	0.952

PPV	**0.629**	0.625

MCC	**0.589**	0.585

(a)		

**HOLO Dataset**	**PLB-SAVE (339 proteins)**	**MPK2 (339 proteins)**

Sensitivity	0.642	**0.806**

Specificity	**0.973**	0.875

Accuracy	**0.949**	0.870

PPV	**0.642**	0.465

MCC	**0.603**	0.561

(b)		

Calculations were performed only on proteins whose binding regions had been successfully predicted by SiteHound and MPK2. Numbers in bold indicate which software gave the better performance for each parameter. (a) PLB-SAVE compared with SiteHound using 374 proteins. (b) PLB-SAVE compared with MPK2 with using 339 proteins.

### Computational performance by CUDA

**Figure 6:**
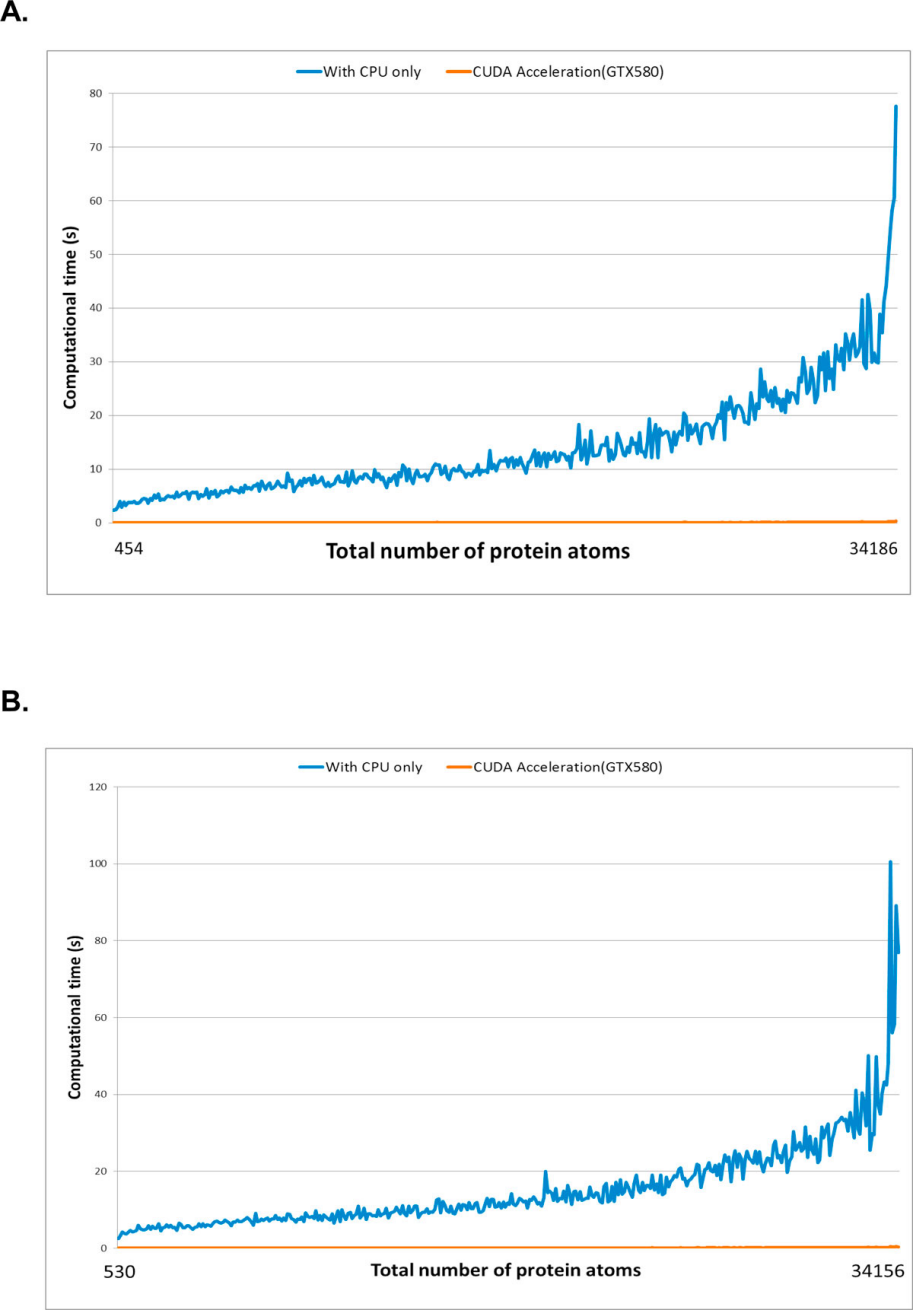
**Required running time for geometric feature computation from both CPU alone and CPU incorporating GPU**. (A) Unbound structure (APO) and (B) bound structure (HOLO) datasets.

The sizes of the 388 unbound protein structures in the APO dataset ranged from 58 to 4520 amino acids, 454 to 34,186 atoms, and 4,510 to 141,201 voxels. The average computational time for computing solid angles with CPU alone and with CUDA acceleration was reduced from 14.1 seconds to 0.088 seconds, respectively. Similarly, the sizes of bound protein structures within complexes in the HOLO dataset ranged from 58 to 4521 amino acids, 530 to 34,156 atoms, and 4,513 to 162,159 voxels. The average computational time for computing solid angles with CPU alone and with CUDA acceleration was reduced from 15.3 seconds to 0.094 seconds, respectively. The relationship between computational time and the total number of atoms in each dataset is shown in Figure 6. Thus, the use of CUDA architecture significantly reduced computational time, and this effect was even more pronounced with increasing protein size, with a nearly 160-fold faster average computation time for test datasets of both bound and unbound protein.

## Conclusions

The use of the geometric construction of solid angles in molecular modeling was originally proposed as early as 1986 by Connolly. It is powerful and is frequently applied to verify the uneven nature of binding surfaces in three-dimensional space. Here, we included consideration of two additional geometric features of the surface anchor residues--depth and volume of the potential cavities-based on their ranked solid angles. We developed an efficient and effective identification system for predicting protein-ligand binding regions using a novel approach based on the combinatorial capabilities of CUDA parallel processing technology. The designed program, PLB-SAVE, included algorithms for calculating solid angles, clustering processes, anchor determination, and derived geometric features. The protein-ligand binding regions identified by PLB-SAVE on protein surfaces were mostly found to have a concave structure based on previous observations. Thus, all possible interactively combined anchors from the query protein can be identified for the potential application of drug and vaccine design strategies. Binding sites between the antibody and antigen are crucial for the efficacy of the protective effect. Recently, carbohydrate-based vaccines have gained increasing attention due to the serotypes of various bacterial or viral strains. As well as the glycans exposed on the surface of cancer cells, carbohydrates have been developed as targets to be neutralized by an antibody or for inducing antibody-dependent cell-mediated cytotoxicity for cancer therapy [42]. Carbohydrate-based vaccines are therefore expected to specifically protect hosts against the infection and eliminate cancer cells by immunotherapy. Thus, prediction of the ligand-binding site, such as a carbohydrate- or a glycan-binding site, would contribute considerably to the field of vaccine development. This research not only emphasizes accurate identification of protein-ligand binding regions, but also provides a practical example of use of the CUDA parallel computing architecture. Two test datasets, which included 388 unbound and bound proteins, were evaluated using our software, PLB-SAVE, and two other well-known programs, SiteHound and MPK2. The results show that our algorithm achieved an average accuracy rate of 95% for correctly identifying protein-ligand binding regions on two unbound and bound proteins, and performed an average of 160 times faster on these test datasets. PLB-SAVE can therefore be used as one of the first prediction tools for protein surface analysis and protein-ligand binding region detection for application in drug and vaccine development.

## Competing interests

The authors declare that they have no competing interests.

## Authors' contributions

YTL and HWW designed the algorithms and performed the computational data analysis. TWP and HTC conceived of the study, participated in its design and coordination and helped draft the manuscript. WST and HHH participated in the design and helped to review the manuscript. All authors read and approved the final manuscript.

## Declarations

The funding for publication of this article is provided by the Center of Excellence for Marine Bioenvironment and Biotechnology in National Taiwan Ocean University and National Science Council, Taiwan, R.O.C.

This article has been published as part of *BMC Bioinformatics *Volume 14 Supplement 4, 2013: Special Issue on Computational Vaccinology. The full contents of the supplement are available online at http://www.biomedcentral.com/bmcbioinformatics/supplements/14/S4
